# Intimate partner violence against women of reproductive age and associated factors during COVID-19 pandemic in Northern Ethiopia, 2021: A community-based cross-sectional study

**DOI:** 10.3389/fgwh.2022.977153

**Published:** 2023-02-07

**Authors:** Mekasha Getnet Demeke, Ehtemariam Tefera Shibeshi

**Affiliations:** ^1^ Department of Nursing, College of Health Sciences, Debre Berhan University, Debre Berhan, Ethiopia; ^2^ Department of Public Health, College of Health Sciences, Debre Berhan University, Debre Berhan, Ethiopia

**Keywords:** COVID-19 pandemic, intimate partner, reproductive age, violence, gender-based violence

## Abstract

**Background:**

Intimate partner violence (IPV) is a major public health concern that affects more than one-third of all women globally. Assessing the prevalence of intimate partner violence and associated factors during the COVID-19 pandemic in various localities is crucial for intervention actions. So far, a few studies have been done in Ethiopia during the current COVID-19 pandemic.

**Objective:**

This study aimed to assess the prevalence of intimate partner violence in women of reproductive age and associated factors during the COVID-19 pandemic in Debre Berhan town, Ethiopia, 2021.

**Methodology:**

A community-based cross-sectional study was done. A total of 809 ever-partnered women of reproductive age were selected randomly *via* a multistage sampling method. Crude and adjusted odds ratios (AOR) with the resulting 95% confidence interval (CI) were used to verify the strength of associations. Significant associations were declared at *p*-values <0.05.

**Result:**

Among the 796 women who successfully participated in the study, 337 (42.3%) experienced at least one type of intimate partner violence. Prevalence of psychological, physical, and sexual violence was 35.3% (281), 15.3% (122), and 15.2% (121), respectively. Multivariate analysis revealed that women with no formal education [AOR (95% CI): 3.66 (1.91–6.98)], having no own income [AOR (95% CI): 1.78 (1.24–2.56)], and attitude of IPV were acceptable [AOR (95% CI): 4.02 (1.33–12.14)]; a male partner with no formal education [AOR (95% CI): 3.06 (1.53–6.14)], with “level of religious beliefs” [weak—AOR (95% CI): 4.17 (1.45–12.03); and medium—AOR (95% CI): 1.64 (1.13–2.39)], who is alcoholic [AOR (95% CI): 5.91 (4.03–8.67)], and with smoking habits [AOR (95% CI): 2.04 (1.10–3.77)] and >5 [AOR (95% CI): 1.83 (1.01–3.39)] was significantly associated with the presence of intimate partner violence.

**Conclusion and recommendation:**

This study revealed a high prevalence of IPV in the study participants. The high intimate partner violence prevalence was due to multiple factors, thus demanding empowering women and tailored health education for male partners.

## 1. Introduction

Gender-based violence (GBV) is any brutality directed at an individual based on their sex, gender identity, or socially defined ways of maleness and femaleness (1–3). Both men and women can experience GBV; however, the rates among women are severely higher (1–4). Thus, violence against women is the primary form of GBV, a major public health problem, and a fundamental violation of women's human rights (1–5). It includes any violent acts such as threats, coercion, and denial of liberty against women (5–7). The actor of violence against women can be anyone, irrespective of their relationship with the victim, whereas the main perpetrators are male partners including husbands, fiancées, or ex-partners, often referred to as intimate partners (5–8).

Intimate partner violence (IPV) is the insidious form of violence against women, which includes physical, sexual, and emotional types of violence (6–10). It has been known that IPV can cause lifelong mental, physical, and reproductive health problems (7, 11–13). Women who experience IPV also risk further conflicts with others and develop social disorders (11–14).

Intimate partner violence occurs among women in developed and developing countries, in all settings, socioeconomic, religious, and cultural groups, without restrictions (1–3). It is estimated that over 35% of women worldwide have experienced IPV at some point in their lives (8–10). In that, nearly 33% of women in a relationship reported having experienced either physical and/or sexual abuse (5–8). However, the rate as well as types of IPV vary across regions, countries, and also among localities within a country (7).

About 27% of women in European and western Pacific regions and 30% of women in South America reported IPV (7–10, 15). The prevalence of IPV was typically high in women across African countries (16–18). For instance, about 50% of women in Côte d'Ivoire experienced IPV (19).

Likewise, a high prevalence of sexual (59%) and physical (49%) violence was reported in women in Ethiopia (7). Evidencing, IPV is a major public health concern affecting the physical, sexual, mental, and social well-being of the women in the country (13, 20–22).

Intimate partner violence increases during conflicts and pandemics (16, 17). During pandemics, people are forced to perform firm protective actions; thus, their normal lifestyles are likely to be changed (23, 24). As the global pandemic of coronavirus (COVID-19) spreads across continents and communities, governments of nearly all countries globally force their people to respond with strict preventive actions such as staying at home, keeping social distance, etc. (20, 24).

A finding from Tajikistan showed that the prevalence of physical, emotional, and sexual violence was 23.2%, 15.5%, and 1.7%, respectively. In this study, the educational level and alcohol-drinking status of husbands were significantly associated with intimate partner violence (71).

The COVID-19 pandemic and the demand for spouses to stay at home can aggravate differences and open up unsettled issues, rising emotive to deficiencies and minor mistakes (25, 26). This pandemic has also been believed to increase risk factors such as alcohol use (25). In acute cases, with a lack of awareness and skills to resolve conflicts, IPV is aggravated and worsens in partners with emotional divorce (25, 27). As well, quarantine, fear of infection, the chaos of social networks, reduced access to health and social services, distress, misinformation, income shortage, financial loss, job loss, and limited social support are likely to raise risks of IPV in the pandemic (25–27). This evidence is supported by a study done in Tajikistan, which stated that intimate partner violence is significantly associated with no or primary educational status and husbands who have alcohol-drinking habits (71).

The government of Ethiopia also affirmed to take several actions since the first cases of COVID-19 were identified in the country (28). As a result, the normal lifestyle of the people across different parts of Ethiopia has been affected notably due to the protective actions, fear of infection, and other socioeconomic effects, which might lead to an increase in the prevalence of IPV in different parts of the country. Different strategies were implemented as prevention measures like homestay, physical distance, washing hands with water and soap, and wearing facemasks (20, 24). Thus, this study aimed to assess the prevalence of IPV in women of reproductive age and the associated risk factors in Debre Berhan town in Ethiopia during the COVID-19 pandemic.

## 2. Methods and materials

### 2.1. Study area

This study was conducted in Debre Berhan, North Shoa Zone of Amhara Region, Ethiopia. Debre Berhan is located about 120 km northeast of the capital city, Addis Ababa, of the country. The town is among the fast-growing cities in Ethiopia. It has a total population of 113,693 (69). Currently, the town has 14 Kebele administrations, among which 9 are urban and the rest are recently included rural kebeles. According to the information obtained from the North Shoa Zone Health Department, there are a total of about 26,663 households in Debre Berhan, which are unevenly distributed throughout the 14 Kebele administrations.

### 2.2. Study design and period

A community-based cross-sectional study design was utilized to assess the prevalence of intimate partner violence in women of reproductive age and associated risk factors in Debre Berhan. The study was conducted from February to April 2021 G.C.

### 2.3. Source population

All women of reproductive age living in Debre Berhan in 2021 G.C were the source population for the present study (Table 1).

**Table 1:** Number of households in each of the kebeles of Debre Berhan, 2021 G.C.

SN	Name of kebele	Locality	HHs per kebele	HHs and kebeles per locality		Remark
1	Kebele 1	Urban	982	22,775 HHs	9 kebeles	
2	Kebele 2	Urban	3,431			Selected
3	Kebele 3	Urban	2,619			Selected
4	Kebele 4	Urban	3,654			
5	Kebele 5	Urban	2,968			
6	Kebele 6	Urban	2,350			
7	Kebele 7	Urban	1,052			Selected
8	Kebele 8	Urban	2,794			Selected
9	Kebele 9	Urban	2,925			
10	Atakilt	Rural	1,569	3,888 HHs	5 kebeles	Selected
11	Zanjira	Rural	578			Selected
12	Chole	Rural	335			
13	Faji	Rural	764			
14	Genet	Rural	642			
Total	26,663	26,663	14	6 kebeles		

HH, household.

Source: The data were obtained from the North Shoa Zone Health Department, Debre Berhan, Ethiopia, 2021.

### 2.4. Study population

Women of reproductive age living in the selected six kebeles of Debre Berhan, namely, Kebele 2, Kebele 3, Kebele 7, Kebele 8, Atakilt, and Zanjira, were the study population for the present study from which the participant women of reproductive age were selected directly.

### 2.5. Inclusion and exclusion criteria

#### 2.5.1. Inclusion criteria

Women of reproductive age who are ever-partnered and living in the selected Kebele during the COVID-19 pandemic since the onset of the COVID-19 pandemic until the data collection period were eligible for this study.

#### 2.5.2. Exclusion criteria

Women of reproductive age who are never-partnered and not living in the selected Kebele during the COVID-19 pandemic and whose partners are not physically with them since the onset of the COVID-19 pandemic until the data collection period were not eligible for this study.

### 2.6. Sample size determination

The sample size for the present study was calculated in harmony with the study objectives using the following two ways. First, the sample size for the prevalence of intimate partner violence (the first objective) was calculated using the single population proportion formula and basic assumptions as shown below:

$$n=\frac{Z^{2}\frac{\alpha }{2}pq}{d^{2}}$$

where

• *n* = desired sample size
• confidence level considered is 95%
• *Z* = standard normal deviate at 95% confidence level (1.96)
• *P* = proportion of IPV prevalence of 24.6% taken from a study conducted previously in Aksum town, northern Ethiopia (20)
• *q* = 1 − *P* (1 − 0.246) = 0.754
• *d* = degree of accuracy desired 5% (0.05).
  ✓ The minimum possible sample size was ≈285.
  ✓ A design effect of 2 was used: 285 × 2 = 570.

Thus, the total sample size calculated considering the 24.6% IPV prevalence was 570 women.

Second, the sample size calculated considering the second objective (regarding the associated factors) was calculated by taking significantly associated factor variables from previous studies that were conducted in Ethiopia and elsewhere (20, 22, 30, 43) by using Epi Info version 7 software *via* the cross-sectional study option, as shown in Table 2.

**Table 2:** Sample size calculation based on factor variables to assess IPV prevalence among women of reproductive age during the COVID-19 pandemic in Debre Berhan, 2021 G.C.

Variables	CI	Power	Unexposed: exposed ratio	IPV prevalence in exposed	IPV prevalence in unexposed	OR	Sample size
Women-related factors							
Age	95	80	0.08	33.2%	7.7%	5.95	380
Occupation	95	80	0.31	45.2%	30.8	1.85	511
Education	95	80	0.48	26.5%	12.7%	2.47	324
Pregnancy status	95	80	5.25	87.2%	28.1%	17.4	40
Acceptance of IPV	95	80	0.49	45.6%	21.4%	3.08	143
Relationship status	95	80	7.68	66.1%	33.8%	3.81	174
Male partner-related factors							
Age	95	80	0.89	30.9%	21.7%	1.61	735
Occupation	95	80	1.89	28.9%	40.1%	1.65	610
Education	95	80	2.4	47.3%	32.9%	1.83	427
Alcohol use	95	80	0.85	53.3%	19%	4.87	64
Smoking	95	80	0.05	82.1%	35.1%	8.5	118

CI, confidence interval; OR, odds ratio; IPV, intimate partner violence.

As shown in Table 2, most of the calculated sample sizes regarding the associated risk factor variables (the second objective of this study) were smaller than the sample size calculated regarding the first objective of this study, equal to 570. However, the sample sizes calculated regarding the age and occupation of the male partners equal to 735 and 610, respectively, were larger than the sample size calculated regarding the first objective of this study (Table 2).

Accordingly, the sample size calculated based on the age of the male partner (equal to 735) was assumed to be optimal for both objectives of this study. Finally, an estimated nonresponse rate of 10% was considered (i.e., 735 × 0.10 = 73.5 ≈ 74), and the final sample size determined for the present study was equal to 735 + 74 = 809 women of reproductive age.

### 2.7. Sampling procedure

In this study, a stratified multistage sampling technique was applied. In the first stage, kebeles (primary sampling units) were selected randomly by a lottery method. At this stage, all of the 14 kebeles in the town were stratified into urban and rural kebeles to have an unbiased allocation of samples between the two localities. After that, nearly half of the kebeles in each of the two localities, which means four of the nine urban kebeles and two of the five rural kebeles, were selected randomly by the lottery method.

In the second stage, households (secondary sampling units) were selected by a systematic random sampling method among the households in each of the six eligible kebeles. At this stage, the list of residents in the respective kebele was used as a sampling frame, while a sampling interval “*K*” was calculated by dividing the number of total households available in a given kebele by the sample size allotted for the kebele.

Finally, one ever-partnered woman of reproductive age was selected per household. In the cases of households where two or more ever-partnered women of reproductive age were available, one of the available ever-partnered women of reproductive age was selected randomly by the lottery method using rolled sheets of “zeros” and “one.” However, in the cases of households where no ever-partnered woman of reproductive age was available, data collectors moved to the next (+1) household until they arrived at a household where an eligible woman was available.

**Figure 1:**
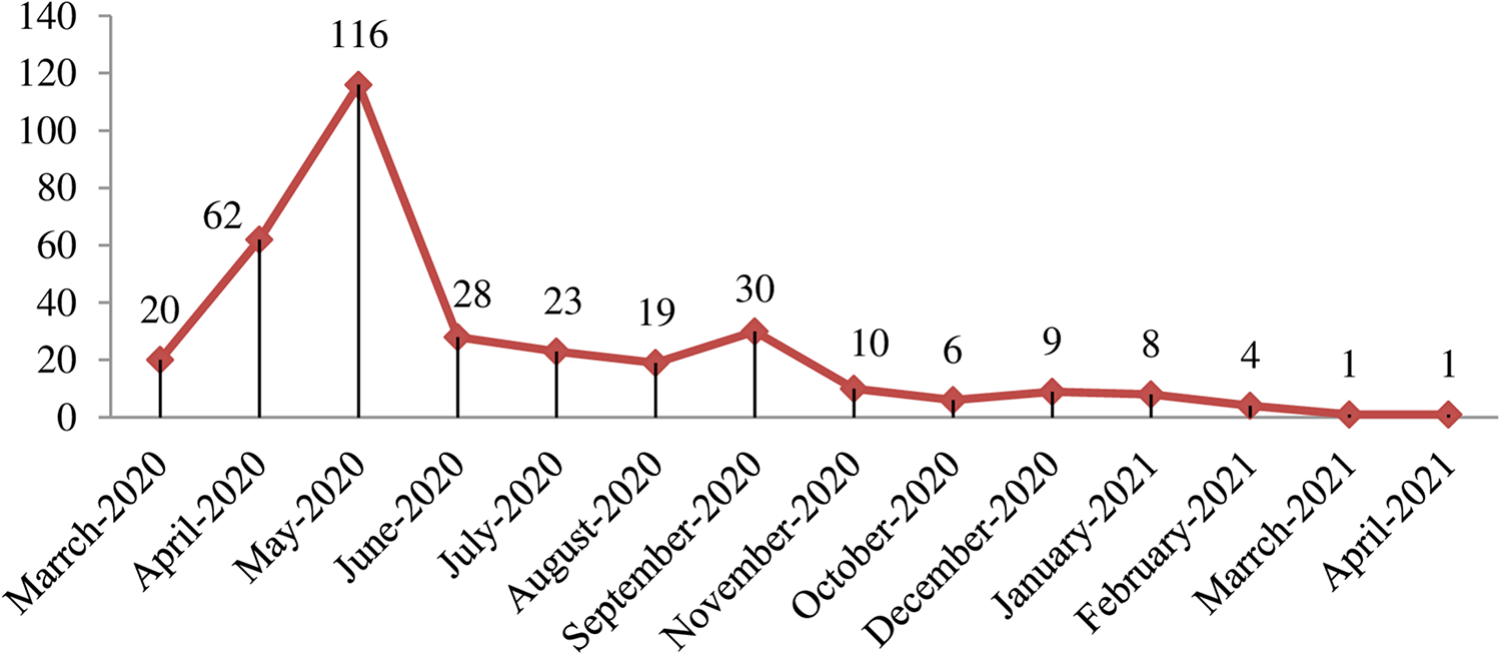
Conceptual framework for factors affecting intimate partner violence against women of reproductive age.

Above all, to ensure an unbiased allotment of samples between rural and urban localities and within the selected kebeles in each locality, numbers of final study units (women of reproductive age) were allotted proportionally to size. Accordingly, using the outlined sampling procedure explained thus far in the text and depicted in the diagram presented beneath, a total of 809 ever-partnered women of reproductive age were enrolled for the present study (Figure 1).

### 2.8. Data collection technique and procedure

**Figure 2:**
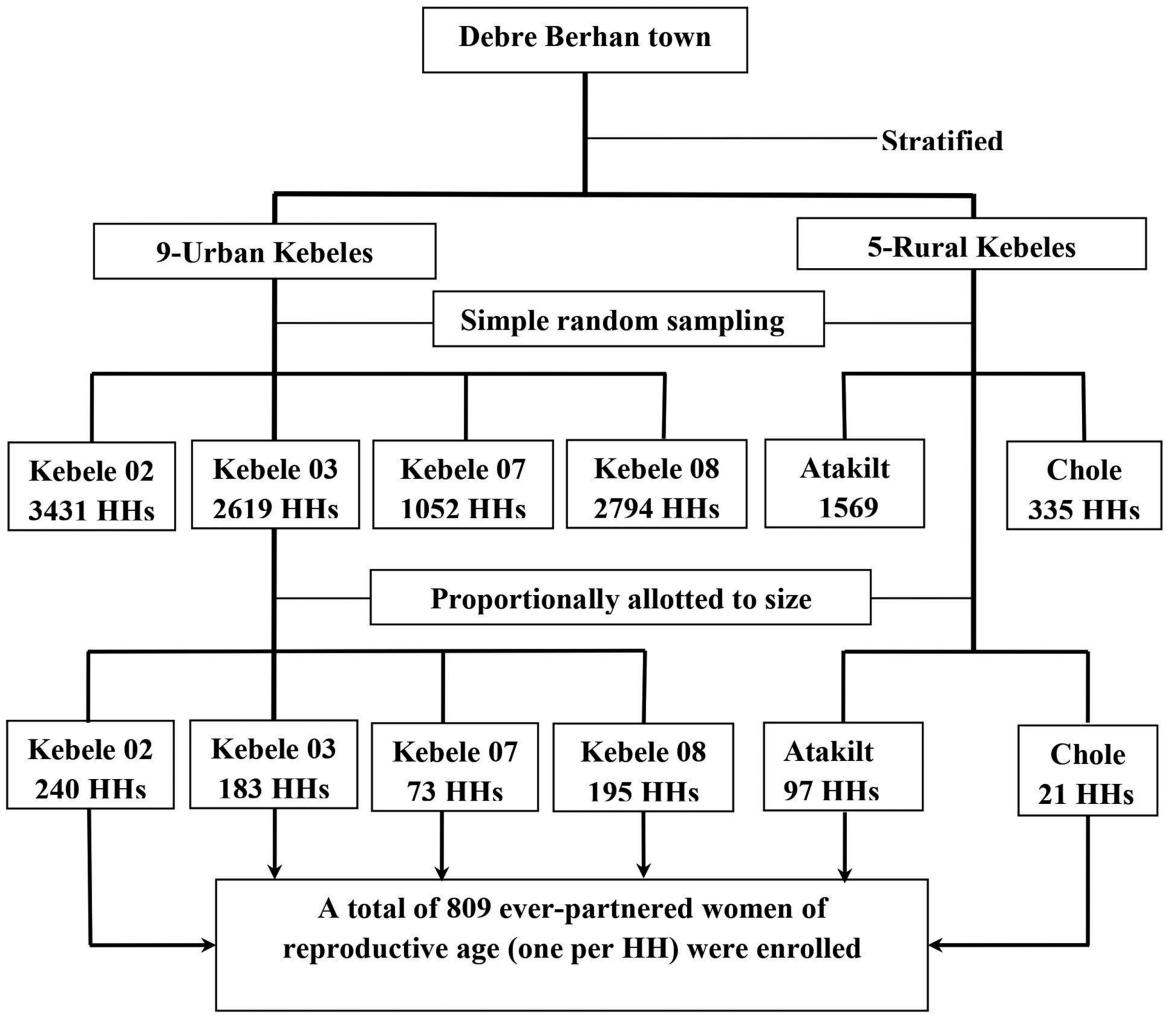
Schematic representation of the sampling procedure of ever partnered women of reproductive age in Debre Berhan town.

Data collection was carried out by using a structured questionnaire set concerning intimate partner violence and the associated factors. The types of intimate partner violence were classified into physical, sexual, and emotional violence, and queries conforming to each type of violence were prepared based on the WHO instrument on violence against women (5). To assess the associated factors, questions regarding women-related, male partner-related, and family-related variables were prepared in line with the conceptual framework outlined in advance (Figure 2).

Finally, each participant ever-partnered woman of reproductive age was asked to complete the questionnaire with the necessary information, either administered by herself or with the help of an interviewer, in accordance with her education status and preference for the procedure.

### 2.9. Variables of the study

#### 2.9.1. Dependent variables

Ø The dependent variable for the present study was any type of IPV.

#### 2.9.2. Independent variables

Ø Independent variables for the present study are as follows:
  • **Women-related factors**: Age, education, occupation, own income, relationship status, religion, access to media (TV/radio), pregnancy status, and acceptance of IPV.
  • **Male partner-related factors**: Age, education, occupation, own income, religious belief (level of attitude rated by the woman), alcohol consumption habit, and smoking habit.
  • **Family-related factors**: Family size, number of children, presence of extended family, and monthly family income.

### 2.10. Operational definitions

• **Acceptance of IPV:** It refers to the attitude or principle of a woman toward the cultural or societal thought of “intimate partner violence is acceptable.”
• **Emotional violence:** It refers to verbal acts such as insults, belittling, humiliation, intimidation like destroying things, threats of harm, and threats to take away children (5).
• **Extended family:** It includes any family member other than the biological (adopted) children of the couple, such as grandparents, parents, uncles, aunts, sisters, brothers, or relatives of the male partner or the woman (47).
• **Intimate partner violence absent:** A woman has not experienced all of the three types of violence (i.e., physical, sexual, and emotional violence) by her intimate partner during the COVID-19 pandemic (i.e., from March 20, 2020, until the data collection period) (5).
• **Intimate partner violence present:** A woman has experienced at least one of the three types of violence (i.e., physical, sexual, and emotional violence) by her intimate partner during the COVID-19 pandemic (i.e., from March 20, 2020, until the data collection period) (5).
• **Intimate partner violence:** Any form of physical, sexual, and emotional violence against women by an intimate partner (5).
• **Intimate partner:** The male partner of the woman in a couple, either her husband (legal or illegal), a fiancée, a boyfriend, or any male sexual partner, who cohabits with the woman (5).
• **Physical violence:** It refers to any of the acts such as slapping, hitting, kicking, and beating against the victim woman by her intimate partner (5).
• **Presence of own income:** The presence of any regular means of income belonging to each individual in a couple (i.e., the woman and the male partner each) (43).
• **Religious belief of male partner:** It refers to the level of attitude or principle of the male partner toward religious faiths, which was measured as rated by the mouth of the woman in rating words, such as weak, medium, and strong.
• **Sexual violence:** It refers to acts including forced sexual intercourse and other forms of sexual bullying against the victim woman by her intimate partner (5).

### 2.11. Data quality control

To ensure data quality, training was given to data collectors and supervisors for 1 day. The questionnaire was administered in Amharic (native language). Before the actual data collection, the questionnaire was tested by taking 5% of the total sample size among women of reproductive age in Debre Sina town. On-spot checks, re-interviewing, and checking completed questionnaires and quality of recordings were done *via* daily supervision by field supervisors. In addition, training was given to all data collectors and supervisors for 2 days before the actual data collection.

### 2.12. Data processing and analysis

Data were entered in Epi.Data Version 4.2 software, while further statistical analyses were done using Statistical Package for Social Sciences (SPSS) version 24. Descriptive statistics were used to describe the prevalence of IPV and sociodemographic characteristics of the study participants in percentages and frequencies. A binary logistic regression model, with bivariate and multivariate analyses, was used to verify the association of each independent variable with the dependent variable.

In the modeling process, first, bivariate logistic regression analysis was performed to detect the association of each independent variable with the dependent variable using a crude odds ratio (COR), 95% confidence interval (CI), and *P*-value. Then, all independent variables with *P*-values ≤ 0.25 in the bivariate analysis were selected and entered in the multivariate logistic regression analysis, while independent variables suspected for collinearity/multicollinearity (coefficients = 0.8) with other variables were excluded (70). Finally, in the multivariate logistic regression analysis, the strength of associations of each independent variable with the dependent variable was verified using an adjusted odds ratio (AOR) and 95% CI. Associations were declared significant at *P*-value < 0.05.

### 2.13. Ethical consideration

Ethical clearance and approval were obtained from the Institutional Review Board of the Institute of Health Science and Medicine, College of Health Science, Debre Berhan University, which was further communicated to zonal and town health departments/offices and to the selected Kebele administrations. Verbal consent was obtained from each participant woman. The names of the study participants were not taken, all the necessary data were collected and registered based on unique codes of women given by the study, and thus all information was kept confidential.

## 3. Results

### 3.1. Sociodemographic characteristics of the study participants

A total of 796 women successfully participated in the study, giving a response rate of 98.4%. Of the 796 study participant women, 332 (41.7%), 368 (46.2%), and 96 (12.1%) were in the age groups of 18–28, 29–38, and 39–45 years, respectively. The age of the male partners of the study participant women ranged from 20 to 68 years. About 115 (14.4%) of women and 77 (9.7%) of male partners were illiterate. The majority of the study participant women (584, 73.4%) were married, 676 (84.9%) had at least one child, and 681 (85.6%) were living in urban kebeles (Table 3).

**Table 3:** Sociodemographic characteristics of the study participant women and their male partners and families in Debre Berhan, Ethiopia, 2021 (*n* = 796).

Variable	Category	Frequency	Percentage
Age of woman (years)	18–28	332	41.7
	29–38	368	46.2
	39–45	96	12.1
Woman education	No formal education	115	14.4
	Primary education	216	27.1
	Junior education	145	18.2
	Secondary education	139	17.5
	Diploma and above	181	22.7
Woman occupation	Farmer	14	1.8
	Manual worker	87	10.9
	Housewife	335	42.1
	Trader/Pettit-trade	210	26.4
	Govt./NGO employee	150	18.8
Woman religion	Orthodox	622	78.1
	Muslim	126	15.8
	Others	48	6.0
Relationship status	Married	584	73.4
	Unmarried	212	26.6
Residence	Rural kebeles	115	14.4
	Urban kebeles	681	85.6
Age of male partner (years)	20–30	246	30.9
	31–40	402	50.5
	41–68	148	18.6
Male partner education	No formal education	77	9.7
	Primary education	199	25.0
	Junior education	123	15.5
	Secondary education	149	18.7
	Diploma and above	248	31.2
Male partner occupation	Farmer	71	8.9
	Manual worker	182	22.9
	Trader/Pettit-trade	227	28.5
	Govt./NGO employee	316	39.7
Family size	3 and below	265	33.3
	4–5	300	37.7
	6 and above	231	29.0
Presence of children	Yes	676	84.9
	No	120	15.1

### 3.2. Prevalence of intimate partner violence in the study participants

Among the 796 study participant women of reproductive age in Debre Berhan, 337 (42.3%) were experiencing at least one type of violence by an intimate partner during the COVID-19 pandemic (Table 4). The prevalence of any form of intimate partner violence in women of reproductive age during the COVID-19 pandemic was 38.3% (44) among women living in rural kebeles and 43% (293) among women living in urban kebeles (Table 4). Also, the overall prevalence of each of the three types of violence, psychological, physical, and sexual violence, in the study participant women of reproductive age in Debre Berhan during the COVID-19 pandemic was 35.3% (281), 15.3% (122), and 15.2% (121), respectively (Table 4).

**Table 4:** Prevalence of intimate partner violence in women of reproductive age in Debre Berhan, Ethiopia, 2021 (*n* = 796).

Type of violence	Total No. (%)	Residence kebeles		*P*-value
		Rural No. (%)	Urban No. (%)	
Psychological violence only	139 (17.5)	22 (19.1)	117 (17.2)	0.621
Physical violence only	5 (0.6)	0 (0.0)	5 (0.7)	0.025
Sexual violence only	49 (6.2)	4 (3.5)	45 (6.6)	0.110
Psychological + physical violence	72 (9.0)	10 (8.7)	62 (9.1)	0.886
Psychological + sexual violence	27 (3.4)	2 (1.7)	25 (3.7)	0.172
Physical + sexual violence	2 (0.3)	0 (0.0)	2 (0.3)	0.157
Psychological + physical + sexual violence	43 (5.4)	6 (5.2)	37 (5.4)	0.924
Overall psychological violence	281 (35.3)	40 (34.8)	241 (35.4)	0.900
Overall physical violence	122 (15.3)	16 (13.9)	106 (15.6)	0.638
Overall sexual violence	121 (15.2)	12 (10.4)	109 (16.0)	0.080
Any IPV (at least one type of IPV) present	337 (42.3)	44 (38.3)	293 (43.0)	0.332
No IPV present	459 (57.7)	71 (61.7)	388 (57.0)	0.332

IPV, intimate partner violence.

Regarding co-occurrence of two or more types of violence, about 72 (9%) of the study participant women were experiencing psychological and physical violence, 27 (3.4%) were experiencing psychological and sexual violence, 2 (0.3%) were experiencing physical and sexual violence, and 43 (5.4%) of the study participants were experiencing all types of violence (psychological, physical, and sexual violence) by an intimate partner during the COVID-19 pandemic (Table 4). However, about 193 (24.3%) of the study participants of reproductive age were experiencing only one type of violence (Table 4). In more detail, about 139 (17.5%) of the study participants were experiencing only psychological violence, 5 (0.6%) were experiencing only physical violence, and 49 (6.2%) were experiencing only sexual violence by intimate partners (Table 4).

**Figure 3:**
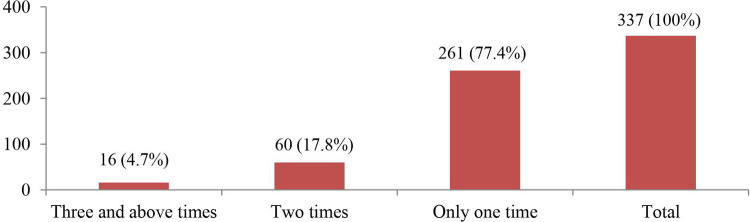
Repetition of IPV experienced by the study participant women of reproductive age in Debre Berhan town, 2021.

Regarding violence repetition on each participant woman across the depth of the study during the pandemic era, among the total of 337 participant women who were experiencing at least one type of IPV, 16 (4.7%) were experiencing violence three and more times, 60 (17.8%) of them were experiencing two times, and the remaining 261 (77.4%) women were experiencing violence only once in the 14 months this study has addressed (Figure 3).

**Figure 4:**
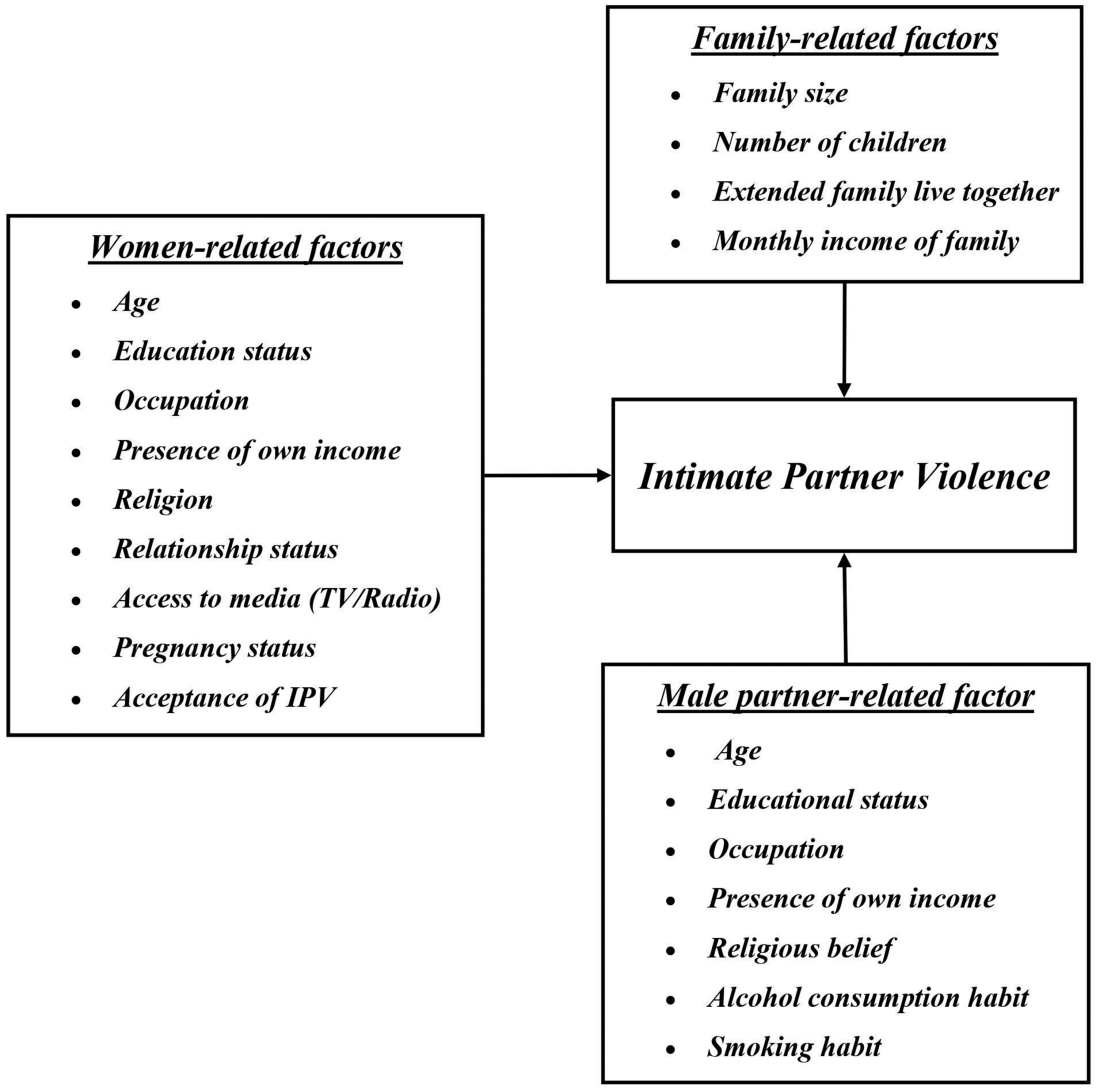
Trend of IPV over the fourteen months prior to the data collection among women of reproductive age in Debre Berhan town, Ethiopia, 2021.

The trend of at least one type of IPV among the study participant women of reproductive age over the 14 months prior to the data collection during the pandemic varied considerably (Figure 4). In the first 3 months of the pandemic, the occurrence of IPV had an increasing trend, with 116 of the total 337 victims experiencing it in May 2020, and the least occurrences of 1 case of IPV occurred during March and April 2021 (Figure 4).

### 3.3. Associated risk factors for intimate partner violence against women

#### 3.3.1. Bivariate logistic regression analysis of associated factors for IPV

The bivariate logistic regression analysis results of the woman-related factors of IPV among the study participant women of reproductive age in Debre Berhan revealed that variables such as the age, education status, own income, and attitude of the woman toward the acceptability of IPV were significantly associated with the presence of any IPV. In addition, according to the bivariate logistic regression analysis results of the male partner-related associated factors, the age, education status, occupation, religious belief, and alcohol-drinking and smoking habits of the male partner showed statistically significant association with the presence of any IPV (Table 6).

In addition to the bivariate logistic regression analyses presented above, a multivariate logistic regression analysis was done for the selected association factor variables for IPV with *P*-values ≤ 0.25 in the bivariate analysis. Accordingly, the multivariate logistic regression analysis showed that the woman-related factor variables, such as woman’s education status of no formal education, primary education, and junior education, showed significant association with the presence of any IPV as evidenced in the results from the AOR with 95% CI with *P*-values of less than 0.001 (AOR = 3.66, 95% CI: 1.91–6.98; AOR = 3.52, 95% CI: 2.04–6.07; and AOR = 3.35, 95% CI: 1.87–6.02), respectively (Tables 5, 8). As well, the risk factor variables such as woman's own income (AOR = 1.78; 95% CI: 1.24–2.56; *P*-value < 0.001), with woman's attitude on IPV acceptance (AOR = 4.02; 95% CI: 1.33–12.14; *P*-value < 0.05), showed significant association with the presence of any IPV (Table 8).

**Table 5:** Bivariate logistic regression analysis of the association of woman-related factors with IPV among women of reproductive age in Debre Berhan town, 2021 (*n* = 796).

Variable	Category	Any IPV		COR (95% CI)	*P*-value
		No Fr (%)	Yes Fr (%)		
Age of woman (years)	18–28	209 (63.0)	123 (37.0)	0.42 (0.27–0.67)	0.000
	29–38	210 (57.1)	158 (42.9)	0.54 (0.34–0.85)	0.008
	39-45	40 (41.7)	56 (58.3)	1
Woman education	No formal education	44 (38.3)	71 (61.7)	7.24 (4.25–12.33)	0.000
	Primary education	97 (44.9)	119 (55.1)	5.50 (3.46–8.74)	0.000
	Junior education	75 (51.7)	70 (48.3)	4.19 (2.54–6.89)	0.000
	Secondary education	95 (68.3)	44 (31.7)	2.08 (1.24–3.49)	0.006
	Diploma and above	148 (81.8)	33 (18.2)	1
Woman occupation	Farmer	9 (64.3)	5 (35.7)	0.73 (0.23–2.27)	0.583
	Manual worker	46 (52.9)	41 (47.1)	1.17 (0.69–1.98)	0.571
	Housewife	188 (56.1)	147 (43.9)	1.02 (0.69–1.51)	0.911
	Trader/Pettit-trade	131 (62.4)	79 (37.6)	0.79 (0.52–1.21)	0.276
	Govt./NGO emp.	85 (56.7)	65 (43.3)	1
Woman religion	Others	30 (62.5)	18 (37.5)	0.85 (0.46–1.55)	0.590
	Muslim	65 (51.6)	61 (48.4)	1.32 (0.90–1.94)	0.152
	Orthodox	364 (58.5)	258 (41.5)	1
Residence	Rural kebeles	71 (61.7)	44 (38.3)	0.82 (0.55–1.23)	0.339
	Urban kebeles	388 (57.0)	293 (43.0)	1
Relation status	Unmarried	118 (55.7)	94 (44.3)	1.12 (0.81–1.54)	0.491
	Married	341 (58.4)	243 (41.6)	1
Woman has own income	No	203 (51.8.)	189 (41.2)	1.61 (1.21–2.14)	0.001
	Yes	256 (63.4)	148 (36.6)	1
Woman access to media	No	33 (50.8)	32 (49.2)	1.35 (0.82–2.25)	0.242
	Yes	426 (58.3)	305 (41.7)	1
Pregnancy status	Pregnant	58 (55.2)	47 (44.8)	1.12 (0.74–1.69)	0.589
	Not pregnant	401 (58.0)	290 (42.0)	1
Woman attitude on IPV	Acceptable	6 (24.0)	19 (76.0)	4.51 (1.78–11.42)	0.001
	Not acceptable	453 (58.8)	328 (41.2)	1

IPV, intimate partner violence; COR, crude odds ratio; CI, confidence interval.

**Table 6:** Bivariate logistic regression analysis of the association of male partner-related factors with IPV among women of reproductive age in Debre Berhan, 2021 (*n* = 796).

Variable	Category	Any IPV		COR (95% CI)	*P*-value
		No Fr (%)	Yes Fr (%)		
Age of male partner (years)	20–30	154 (62.6)	92 (37.4)	0.54 (0.36–0.81)	0.003
	31–40	235 (58.5)	167 (41.5)	0.64 (0.44–0.93)	0.020
	41– 68	70 (47.3)	78 (52.7)	1
Male partner education	No formal education	25 (32.5)	52 (67.5)	5.08 (2.93–8.81)	0.000
	Primary education	90 (45.2)	109 (54.8)	2.96 (2.00–4.38)	0.000
	Junior education	73 (59.3)	50 (40.7)	1.67 (1.07–2.63)	0.026
	Secondary education	95 (63.8)	54 (36.2)	1.39 (0.90–2.14)	0.136
	Diploma and above	176 (71.0)	72 (29.0)	1
Male partner occupation	Farmer	37 (52.1)	34 (47.9)	1.61 (0.96–2.70)	0.074
	Manual worker	93 (51.1)	89 (48.9)	1.67 (1.16–2.42)	0.006
	Trader/Pettit-trade	128 (56.4)	99 (43.6)	1.35 (0.95–1.92)	0.090
	Govt./NGO employee	201 (63.6)	115 (36.4)	1
Male partner own income	No	23 (46.9)	26 (53.1)	1.59 (0.89–2.83)	0.119
	Yes	436 (58.4)	311 (41.6)	1
Religious belief of the male partner	Weak	7 (23.3)	23 (76.7)	7.36 (3.04–17.78)	0.000
	Medium	255 (53.0)	226 (47.0)	1.98 (1.46–2.70)	0.000
	Strong	197 (69.1)	88 (30.9)	1
Alcoholic habit of the male partner	Yes	77 (28.8)	190 (71.2)	6.41 (4.63–8.88)	0.000
	No	382 (72.2)	147 (27.8)	1
Smoking habit of the male partner	Yes	20 (24.4)	62 (75.6)	4.95 (2.92–8.38)	0.000
	No	439 (61.5)	275 (38.5)	1

IPV, intimate partner violence; COR, crude odds ratio; CI, confidence interval.

**Table 7:** Bivariate logistic regression analysis of the association of family-related factors with IPV among women of reproductive age in Debre Berhan, 2021 (*n* = 796).

Variable	Category	Any IPV		COR (95% CI)	*P*-value
		No Fr (%)	Yes Fr (%)		
Family size	≤3	179 (67.5)	86 (32.5)	1
	4–5	167 (55.7)	133 (44.3)	1.66 (1.18–2.34)	0.004
	≥ 6	113 (48.9)	118 (51.1)	2.17 (1.51–3.13)	0.000
Presence of children	Yes	372 (55.0)	304 (45.0)	2.15 (1.40–3.31)	0.000
	No	87 (72.5)	33 (27.5)	1
Number of children	None	87 (72.5)	33 (27.5)	1
	Single	128 (64.3)	71 (35.7)	1.46 (0.89–2.40)	0.132
	2–4	238 (51.5)	224 (48.5)	2.48 (1.60–3.85)	0.000
	≥5	6 (40.0)	9 (60.0)	3.96 (1.31–11.98)	0.015
Extended family living together	Present	211 (54.5)	176 (45.5)	1.29 (0.97–1.70)	0.081
	Not present	248 (60.6)	161 (39.4)	1
Monthly family income in ETB	≤1,000	67 (56.8)	51 (43.2)	0.76 (0.34–1.70)	0.505
	1,001–3,500	175 (59.3)	120 (40.7)	0.69 (0.32–1.46)	0.326
	3,501–6,000	138 (58.2)	99 (41.8)	0.72 (0.34–1.54)	0.392
	6,001–10,000	64 (55.2)	52 (44.8)	0.81 (0.36–1.82)	0.613
	>10,000	15 (50.0)	15 (50.0)	1

IPV, intimate partner violence; COR, crude odds ratio; CI, confidence interval; ETB, Ethiopian birr.

**Table 8:** Bivariate and multivariate logistic regression analysis of the association of selected risk factors with IPV among women of reproductive age in Debre Berhan, 2021 (*n* = 796).

Variable	Category	OR (95% CI)	
		Crude	Adjusted
Age of woman (years)	18–28	0.42 (0.27–0.67)**	0.88 (0.38–2.04)
	29–38	0.54 (0.34–0.85)**	0.90 (0.44–1.82)
	39–45	1	1
Woman education	No formal education	7.24 (4.25–12.33)**	3.66 (1.91–6.98)**
	Primary education	5.50 (3.46–8.74)**	3.52 (2.04–6.07)**
	Junior education	4.19 (2.54–6.89)**	3.35 (1.87–6.02)**
	Secondary education	2.08 (1.24–3.49)**	1.79 (0.97–3.28)
	Diploma and above	1	1
Woman has own income	No	1.61 (1.21–2.14)**	1.78 (1.24–2.56)**
	Yes	1	1
Woman access to media	No	1.35 (0.82–2.25)	0.98 (0.51–1.88)
	Yes	1	1
Woman attitude on IPV	Acceptable	4.51 (1.78–11.42)**	4.02 (1.33–12.14)*
	Not acceptable	1	1
Age of the male partner (years)	20–30	0.54 (0.36–0.81)**	1.03 (0.48–2.21)
	31–40	0.64 (0.44–0.93)*	0.88 (0.48–1.61)
	41-68	1	1
Male partner education status	No formal education	5.08 (2.93–8.81)**	3.06 (1.53–6.14)**
	Primary education	2.96 (2.00–4.38)**	1.46 (0.85–2.50)
	Junior education	1.67 (1.07–2.63)*	0.93 (0.51–1.68)
	Secondary education	1.39 (0.90–2.14)	0.91 (0.52–1.61)
	Diploma and above	1	1
Male partner occupation	Farmer	1.61 (0.96–2.70)	0.50 (0.24–1.02)
	Manual worker	1.67 (1.16–2.42)**	0.95 (0.56–1.62)
	Trader/Pettit-trade	1.35 (0.95–1.92)	0.76 (0.48–1.22)
	Govt./NGO employee	1	1
Male partner has own income	No	1.59 (0.89–2.83)	1.82 (0.85–3.92)
	Yes	1	1
Religious belief of the male partner	Weak	7.36 (3.04–17.78)**	4.17 (1.45–12.03)**
	Medium	1.98 (1.46–2.70)**	1.64 (1.13–2.39)*
	Strong	1	1
Alcohol habit of the male partner	Yes	6.41 (4.63–8.88)**	5.91 (4.03–8.67)**
	No	1	1
Smoking habit of the male partner	Yes	4.95 (2.92–8.38)**	2.04 (1.10–3.77)*
	No	1	1
Family size	3 and below	1	1
	4–5	1.66 (1.18–2.34)**	1.73 (1.03–2.92)*
	6 and above	2.17 (1.51–3.13)**	1.85 (1.01–3.39)*
Presence of children	Yes	2.15 (1.40–3.31)**	1.39 (0.74–2.61)
	No	1	1
Number of children**^a^**	No child	1	—
	Single child	1.46 (0.89–2.40)	—
	2–4 children	2.48 (1.60–3.85)**	—
	≥5 children	3.96 (1.31–11.98)*	—
Extended family living together	Present	1.29 (0.97–1.70)	1.05 (0.70–1.59)
	Not present	1	1

IPV, intimate partner violence; OR, odds ratio; CI, confidence interval.

^a^ Variable(s) not included in the multivariate analysis due to collinearity with other variable(s).

* Significant at *P* < 0.05; **Significant at *P* < 0.01.

Likewise, the multivariate logistic regression analysis results showed that the male partner-related factor variables such as lack of formal education (AOR = 3.06; 95% CI: 1.53–6.14; *P*-value < 0.001), weak (AOR = 4.17; 95% CI: 1.45–12.03; *P*-value < 0.001) and medium levels of religious belief (AOR = 1.64; 95% CI: 1.13–2.39; *P*-value < 0.05), alcohol-drinking habit of the male partner (AOR = 5.91; 95% CI: 4.03–8.67; *P*-value < 0.001), and smoking habit (AOR = 2.04; 95% CI: 1.10–3.77; *P*-value < 0.05) of the male partner showed significant association with the presence of any IPV (Tables 6, 8).

Regarding the family-related risk factor variables, the multivariate logistic regression analysis results showed that the family size of four to five members (AOR = 1.73; 95% CI: 1.03–2.92; *P*-value < 0.05) and family size of more than five members (AOR = 1.83; 95% CI: 1.01–3.39; *P*-value < 0.05) showed significant association with the presence of any IPV (Tables 7, 8). In contrast, factors such as the age of the woman, the age of the male partner, occupation of the male partner, and the presence of children did not show significant association with the presence of any IPV, considering *P*-values of < 0.05, regardless of the level of association each of these variables showed in the bivariate analysis (Tables 7, 8).

#### 3.3.2. Analysis of risk factors associated with each of the three types of violence

In addition to the analyses of the risk factors associated with the presence of any IPV (at least one of the three types of violence) as presented above, the present study also attempted to analyze risk factors associated with each of the three types of violence psychological, physical, and sexual violence separately. Similar statistical procedures were applied. The separate analysis results are explicitly presented in the supplementary annex 5.

At the same time, analysis results for the factors that had significant association with each type of violence are presented in the text and a table herewith. Accordingly, the education status of women below a diploma, a woman with the occupation of farmer and housewife, a woman with no own income, a woman with the thought that IPV is acceptable, a male partner without formal education, and a male partner with alcohol-drinking habit were significant predictors of psychological violence (Table 9).

**Table 9:** Bivariate and multivariate logistic regression analyses of the association of factors with each type of IPV among women of reproductive age in Debre Berhan, 2021 (*n* = 796).

Variable	Category	Violence		*P*-value	COR (95%CI)	*P*-value	AOR (95% CI)
		No	Yes				
Psychological violence							
Woman education	No formal education	59	56	0.000	5.66 (3.25–9.84)	0.001	3.26 (1.66–6.38)
	Primary education	109	107	0.000	5.85 (3.57–9.59)	0.000	4.92 (2.73–8.87)
	Junior education	89	56	0.000	3.75 (2.20–6.39)	0.000	3.28 (1.76–6.13)
	Secondary education	103	36	0.011	2.08 (1.19–3.66)	0.028	2.07 (1.08–3.96)
	Diploma and above	155	26	1		1	
Woman occupation	Farmer	13	1	0.066	0.15 (0.02–1.14)	0.001	0.02 (0.01–0.20)
	Labor worker	51	36	0.303	1.33 (0.77–2.29)	0.052	0.47 (0.22–1.01)
	Housewife	213	122	0.710	1.08 (0.72–1.62)	0.004	0.38 (0.20–0.74)
	Trader/petit-trade	140	70	0.792	0.94 (0.61–1.47)	0.373	0.77 (0.44–1.36)
	Govt./NGO employee	98	52	1		1	
Woman has own income	No	280	124	0.006	1.51 (1.13–2.02)	0.000	2.79 (1.71,4.56)
	Yes	235	157	1		1	
Male partner education	No formal education	34	43	0.000	3.56 (2.09–6.06)	0.033	2.13 (1.06–4.28)
	Primary education	109	90	0.000	2.33 (1.56–3.46)	0.639	1.14 (0.66–1.99)
	Junior education	86	37	0.432	1.21 (0.75–1.95)	0.316	0.73 (0.39–1.35)
	Secondary education	103	46	0.317	1.26 (0.80–1.97)	0.427	0.79 (0.43–1.42)
	Diploma and above	183	65	1		1	
Alcohol habit of the male partner	Yes	98	169	0.000	6.42 (4.64–8.88)	0.000	6.31 (4.30–9.27)
	No	417	112	1		1	
Physical violence							
Woman education	No formal education	92	23	0.000	8.80 (3.24–23.91)	0.007	4.49 (1.52–13.31)
	Primary education	169	47	0.000	9.79 (3.80–25.21)	0.001	5.66 (2.05–15.65)
	Junior education	117	28	0.000	8.42 (3.16–22.44)	0.001	6.17 (2.15–17.70)
	Secondary education	120	19	0.001	5.57 (2.03–15.33)	0.002	5.44 (1.85–16.00)
	Diploma and above	176	5	1		1	
Woman has own income	No	323	69	0.080	1.42 (0.96–2.09)	0.020	1.76 (1.09–2.84)
	Yes	351	53	1		1	
Alcohol habit of the male partner	Yes	177	90	0.000	7.90 (5.09–12.24)	0.000	5.58 (3.46–8.99)
	No	497	32	1		1	
Smoking habit of the male partner	Yes	42	40	0.000	7.34 (4.50–11.99)	0.000	4.46 (2.51–7.95)
	No	632	82	1		1	
Sexual violence							
Woman education	No formal education	87	28	0.000	4.53 (2.20–9.35)	0.194	1.77 (0.76–4.17)
	Primary education	180	36	0.003	2.82 (1.42–5.59)	0.377	1.43 (0.65–3.13)
	Junior education	115	30	0.000	3.67 (1.81–7.47)	0.005	3.19 (1.42–7.14)
	Secondary education	124	15	0.188	1.70 (0.77–3.77)	0.447	1.41 (0.58–3.44)
	Diploma and above	169	12	1		1	
Woman has own income	No	355	49	0.015	1.63 (1.10–2.42)	0.001	2.21 (1.37–3.57)
	Yes	320	72	1		1	
Woman access to media	No	47	18	0.004	2.34 (1.31–4.18)	0.007	2.83 (1.33–6.02)
	Yes	628	103	1		1	
Attitude of women toward IPV	Acceptable	13	12	0.000	5.61 (2.49–12.61)	0.000	7.35 (2.76–19.62)
	Not acceptable	662	109	1		1	
Male partner education	No formal education	50	27	0.000	7.34 (3.72–14.47)	0.000	4.90 (2.13–11.25)
	Primary education	162	37	0.000	3.10 (1.69–5.70)	0.387	1.40 (0.65–3.02)
	Junior education	105	18	0.018	2.33 (1.16–4.70)	0.759	1.14 (0.50–2.60)
	Secondary education	127	22	0.012	2.35 (1.21–4.60)	0.419	1.39 (0.63–3.08)
	Diploma and above	231	17	1		1	
Religious belief of the male partner	Weak	19	11	0.000	6.92 (2.93–16.37)	0.018	3.41 (1.24–9.37)
	Medium	393	88	0.000	2.68 (1.64–4.38)	0.001	2.46 (1.42–4.29)
	Strong	263	22	1		1	
Smoking habit of the male partner	Yes	53	29	0.000	3.70 (2.24–6.12)	0.003	2.48 (1.35–4.55)
	No	622	92	1		1	
Family size	≤3	237	28	1		1	
	4–5	254	46	0.095	1.53 (0.93–2.53)	0.039	2.08 (1.04–4.17)
	≥ 6	184	47	0.003	2.16 (1.30–3.59)	0.003	3.01 (1.44–6.29)

IPV, intimate partner violence; COR, crude odds ratio; AOR, adjusted odds ratio; CI, confidence interval.

Likewise, a woman with an education below diploma, a woman without income, and male partners with alcohol-drinking and smoking habits were the significant predictors of physical violence (Table 9). Also, a woman with junior education, no own income, thought of IPV as acceptable, and no access to media, male partners without formal education, strong religious beliefs, and smoking habits, and larger family sizes were the main predictors of sexual violence (Table 9).

## 4. Discussion

Investigation of the prevalence of IPV in women of reproductive age and associated factors in various localities of a given country, particularly during emergencies, including pandemics, is vital for intervention strategies. Accordingly, the present study assessed the prevalence of IPV in women of reproductive age and associated factors in Debre Berhan during the COVID-19 pandemic.

The present study revealed a high overall prevalence of IPV (42.3%) in the study participant women of reproductive age in Debre Berhan during the COVID-19 pandemic. This figure was significantly higher than the overall prevalence of IPV (24.6%) revealed by a previous study conducted in Aksum town, Ethiopia, during the COVID-19 pandemic (20). This finding was also significantly higher compared with 29%, 30%, 37.1%, and 37.5% lifetime prevalence of IPV in women of reproductive age reported in the Amhara region (29), nationwide (30), Debre Tabor town (47), and in the Tigray district (22), respectively, before the pandemic. The present finding was equivalent to the overall IPV prevalence of 40%–50% in Brazil during the COVID-19 pandemic (25), 48% lifetime IPV prevalence in Saudi Arabia (43), and 40.9%–45.2% in Zimbabwe (46). The disparities observed in the overall prevalence of IPV among studies might be due to variations in sociodemographic characteristics of the study participant women, education status of the male partners, addiction status of the male partner, family-related risk factors, duration of data collection periods, and variations in reactions of the communities to the pandemic.

In addition, the present study revealed the highest prevalence of psychological violence (35.3%) among all three forms of IPV, followed by physical (15.3%) and sexual (15.3%) violence. Regarding the ranks within the three types of violence, the present study finding was consistent with results from previous studies conducted in Ethiopia and elsewhere (15, 20, 43). Concerning the prevalence of each of the three types of violence, psychological, physical, and sexual violence, the present study found higher prevalence of IPV in each of the respective types of violence than those revealed by a study done in Aksum town, Ethiopia (20) and a study conducted in Vitória, State of Espírito Santo, Brazil (15). In contrast to the present findings of the prevalence of each of the three types of IPV, higher lifetime prevalence of psychological (48.5%), physical (34.8%), and sexual (16.8%) violence was reported by a study done in Saudi Arabia before the COVID-19 pandemic (43).

In addition to the understanding of the overall prevalence of any IPV and each of the three types of violence, a systematic investigation of the significant factors associated with the presence of IPV is fundamental for enhanced intervention strategies. For that reason, the present study also tried to assess the factors associated with the presence of any IPV (at least one type of violence).

According to the results of the present study, the women-related factors such as woman’s education status, own income, and attitude toward acceptance of IPV were strongly associated with the presence of any IPV among women of reproductive age in Debre Berhan.

The chance of having at least one type of IPV was about 3.7 times higher in women who have no formal education, 3.5 times higher in women who have primary education, and 3.4 times higher in women who have junior education compared with the women who have a diploma or above. The present finding on the significant association of lower education status of a woman with the presence of IPV was consistent with the findings of previous studies done in several parts of Ethiopia (29, 30, 48) and elsewhere (15). This might be due to the lower awareness of less educated women to refuse IPV and guard themselves by the law or other ways.

Regarding the other woman-related factors that showed significant association with the presence of any IPV among the present study participants, the likelihood of experiencing at least one type of IPV was about 1.8 times higher among women who reported they lacked income compared with the women who reported having their own income. The present finding on the significant association of the lack of own income of women with the presence of any IPV was consistent with the findings of previous studies done in Ethiopia (30), Zimbabwe (46), and Brazil (15). This might be due to the fact that women who are economically dependent on male partners or are not self-reliant have insufficient capacity to defend themselves from such troubles.

The attitude of women regarding the acceptability of IPV is the most important women-related risk factor that showed a strong significant association with the presence of any IPV. Given that, the chance of experiencing at least one type of IPV was about four times higher among women who reported IPV as acceptable compared with those women who reported IPV as not acceptable. This finding was in agreement with the findings of previous studies conducted on women in Ofla district (22), Debre Tabor town, Ethiopia (66), and Uganda (65). This might be related to the fact that women who think IPV is acceptable are less likely to refuse violence against them by male partners, which might further enable male partners to view the violence they do against women partners as a normal act rather than a taboo.

In the same way, the present study revealed the male partner-related risk factor variables, such as low educational status, poor religious beliefs, alcohol consumption, and smoking habit of male partners, were significantly associated with the presence of any IPV.

The odds of having at least one type of IPV were about 3.1 times higher in women whose partners have no formal education than women whose male partners have a diploma or above. The present finding regarding the significant association of lower education status of male partners with the presence of IPV against women was in harmony with the findings of previous studies done in Brazil (15, 67), Sudan (45), and Ethiopia (29). This might be due to a poorer tendency to handle conditions that may lead to violence among less educated male partners.

The likelihood of experiencing at least one type of IPV was about 4.2 times and 1.6 times higher in women whose male partners have weak and medium levels of religious beliefs, respectively, compared to women whose male partners have strong religious beliefs. Even if there are theoretical frames that showed the connection between the level of religious beliefs with the attitude of male partners toward taking violent actions against women, empirical evidence reporting the significance of the association of the variable with the occurrence of IPV is rare.

The chance of experiencing at least one type of IPV among women whose male partners have alcohol consumption habits was about 5.9 times higher than women whose male partners are nonalcoholic. The present study finding on the significant association of alcohol consumption habits of male partners with the presence of IPV against women was consistent with previous studies done in Brazil (15, 67), Nigeria (31), Sudan (45), and different parts of Ethiopia (22, 29, 48).

The odds of having at least one type of IPV in women whose male partners have smoking habits were about two times higher than their counterparts. The present finding on the significant association of the smoking habit of male partners with the presence of IPV against women was consistent with the finding of a previous study done elsewhere (67). The significant associations of the above drug use habits of male partners with the presence of any IPV can be explained by the effect the chemicals in the aforesaid drugs can cause on the functioning of the brain of users and by the social and economic consequences of drug addiction.

Regarding the family-related risk factor variables, the present study revealed that family size was the only association factor significantly associated with the presence of any IPV among the present study participant women of reproductive age in Debre Berhan during the COVID-19 pandemic.

Accordingly, the chances of having at least one type of IPV were about 1.7 and 1.8 times higher among women with family sizes of 4–5 and above 5, respectively, compared with those with a family size of less than 4. This finding of the present study can be explained by the resource and other economic issues or limitations often linked to a large family size, which might aggravate the occurrence of IPV (47). However, empirical evidence that revealed the significant association of the risk factor with the presence of IPV is rare. The study was done by using standardized questionnaires, assessing IPV specifically during the COVID-19 pandemic, and using a wider time frame to collect data, including ever-partnered rather than ever-married women. However, the present study has not supported the quantitative finding with qualitative triangulation.

## 5. Recommendations and conclusions

The present study revealed a high overall prevalence of IPV in the study participant women of reproductive age in Debre Berhan during the COVID-19 pandemic, which evidences a major public health significance that needs critical attention. The high overall prevalence of any IPV in the study participants proves that about nine of every twenty women of reproductive age in the town are at risk of experiencing at least one of the three types of IPV during the COVID-19 pandemic.

Regarding the prevalence of each of the three types of IPV in women, the present study disclosed that psychological violence was the most prevalent type of IPV, followed by physical and sexual violence, among the study participant women of reproductive age in Debre Berhan during the COVID-19 pandemic. The study showed that about 7 of every 20 women of reproductive age in the town are at risk of having psychological violence, about 3 of every 20 women of reproductive age in the town are at risk of having physical violence, and about 3 of every 20 women of reproductive age in the town are at risk of experiencing sexual violence by an intimate partner during the COVID-19 pandemic.

Statistical analyses revealed that the high prevalence of any IPV in the study participant women of reproductive age in Debre Berhan during the COVID-19 pandemic was significantly associated with multiple risk factors related to women, male partners, and families. The woman-related risk factors of woman’s lower educational status, lack of own income, and attitude toward IPV as acceptable; the male partner-related risk factors of male partner’s lower educational status, poor religious beliefs, and alcohol-drinking and smoking habits; and the family-related risk factors of large family size were significantly associated with the presence of any IPV among the present study participant women of reproductive age in Debre Berhan during the COVID-19 pandemic.

Based on the main findings of the present study, the following recommendations have been given to the respective bodies.

In making decisions and in planning to tackle the problem of IPV in the long run, policymakers should take into account the need to address the main risk factors significantly predicting the presence of any IPV. As a result, ways to improve the educational status of girls (women), empower women economically, and provision of tailored health education programs regarding the miss-thoughts of women, such as the attitude of accepting IPV, should be devised. Policymakers should also make ways of identifying and tackling the coexisting consequences such as IPV in settings where pandemics are occurring.

In addition, whenever policymakers are working on future attempts to recover some of the social, economic, and health impacts of the COVID-19 pandemic across communities, they should also take into account the high prevalence of IPV against women in different parts of Ethiopia, including the present study area, Debre Berhan ,and the long-lasting effect that IPV causes on its victims.

The North Shoa Zone and Debre Berhan health offices should work in collaboration with other sectors that are working on gender-related issues in the zone and the town regarding various issues related to women empowerment and family planning and also work toward the diffusion of information and concepts on the existing laws that may help the women to protect themselves from violent acts of male partners.

Further researchers should conduct prevalence studies in different localities of the country where data regarding the prevalence of IPV and associated risk factors during the current COVID-19 pandemic are not available. In addition, future researchers should also try to integrate qualitative study methods and more specific variables that can directly measure the impact of the COVID-19 pandemic (or future pandemics) on each of the study units.

## Data Availability

The raw data supporting the conclusions of this article will be made available by the authors, without undue reservation.
