# Supplementary material for: Intimate partner violence against women of reproductive age and associated factors during COVID-19 pandemic in Northern Ethiopia, 2021: A community-based cross-sectional study
Source: Front Glob Womens Health. 2023 Feb 7;3:977153. doi: 10.3389/fgwh.2022.977153 (PMC9941189; doi:10.3389/fgwh.2022.977153)
Supplement: Supplementary file 1 [file Table1.docx]

S1 Supplementary file

**Bivariate and multivariate logistic regression analysis factors for psychological violence**

| **Variable** | **Category** | **Violence** | | **P-value** | **COR(95%CI)** | **P-value** | **AOR(95%CI)** |
| --- | --- | --- | --- | --- | --- | --- | --- |
|  |  | **No** | **Yes** |  |  |  |  |
| Age of women in years | 18 to 28 | 232 | 100 | 0.001 | 0.50(0.28,0.72) | 0.978 | 0.99(0.42,2.33) |
|  | 29 to 38 | 234 | 134 | 0.026 | 0.60(0.38, 0.94) | 0.960 | 0.98(0.48,2.02) |
|  | 39 to 45 | 49 | 47 | 1 | | 1 | |
| Woman education | No formal education | 59 | 56 | 0.000 | 5.66(3.25, 9.84) | 0.001 | 3.26(1.66,6.38) |
|  | Primary educ. | 109 | 107 | 0.000 | 5.85(3.57, 9.59) | 0.000 | 4.92(2.73,8.87) |
|  | Junior educ. | 89 | 56 | 0.000 | 3.75(2.20, 6.39) | 0.000 | 3.28(1.76,6.13) |
|  | Secondary educ. | 103 | 36 | 0.011 | 2.08(1.19, 3.66) | 0.028 | 2.07(1.08,3.96) |
|  | Diploma & above | 155 | 26 | 1 | | 1 | |
| Woman occupation | Farmer | 13 | 1 | 0.066 | 0.15(0.02, 1.14) | 0.001 | 0.02(0.01,0.20) |
|  | Labor worker | 51 | 36 | 0.303 | 1.33(0.77, 2.29) | 0.052 | 0.47(0.22,1.01) |
|  | Housewife | 213 | 122 | 0.710 | 1.08(0.72, 1.62) | 0.004 | 0.38(0.20,0.74) |
|  | Trader/petit-trade | 140 | 70 | 0.792 | 0.94(0.61, 1.47) | 0.373 | 0.77(0.44,1.36) |
|  | Govt/NGO employee | 98 | 52 | 1 | | 1 | |
| Own income of woman | No | 280 | 124 | 0.006 | 1.51(1.13, 2.02) | 0.000 | 2.79(1.71,4.56) |
|  | Yes | 235 | 157 | 1 | | 1 | |
| Age of male partner | 20 to 30 years | 173 | 73 | 0.006 | 0.55(0.36, 0.85) | 0.984 | 0.99(0.45,2.17) |
|  | 31 to 40 years | 258 | 144 | 0.112 | 0.73(0.50, 1.08) | 0.890 | 0.96(0.52,1.77) |
|  | 41 to 68 years | 84 | 64 | 1 | | 1 | |
| Male partner education | No formal education | 34 | 43 | 0.000 | 3.56(2.09, 6.06) | 0.033 | 2.13(1.06,4.28) |
|  | Primary educ. | 109 | 90 | 0.000 | 2.33(1.56, 3.46) | 0.639 | 1.14(0.66,1.99) |
|  | Junior educ. | 86 | 37 | 0.432 | 1.21(0.75, 1.95) | 0.316 | 0.73(0.39,1.35) |
|  | Secondary educ. | 103 | 46 | 0.317 | 1.26(0.80, 1.97) | 0.427 | 0.79(0.43,1.42) |
|  | Diploma & above | 183 | 65 | 1 | | 1 | |
| Male partner occupation | Farmer | 41 | 30 | 0.062 | 1.65(0.97, 2.80) | 0.387 | 1.38(0.67,2.86) |
|  | Labor worker | 106 | 76 | 0.013 | 1.62(1.11, 2.37) | 0.175 | 1.46(0.85,2.50) |
|  | Trader/petit-trade | 149 | 78 | 0.368 | 1.18(0.82, 1.70) | 0.548 | 0.86(0.53,1.40) |
|  | Govt/NGO employee | 219 | 97 | 1 | | 1 | |
| Own income of male partner | No | 487 | 260 | 0.255 | 1.41(0.78, 2.52) | 0.255 | 1.41(0.78,2.52) |
|  | Yes | 28 | 21 | 1 | | 1 | |
| Religious belief of male partner | Weak | 14 | 16 | 0.003 | 3.14(1.46, 6.75) | 0.104 | 2.18(0.85,5.58) |
|  | Medium | 292 | 189 | 0.000 | 1.78(1.29, 2.45) | 0.157 | 1.33(0.90,1.97) |
|  | Strong | 209 | 76 | 1 | | 1 | |
| Alcoholics habit of male partner | Yes | 98 | 169 | 0.000 | 6.42(4.64, 8.88) | 0.000 | 6.31(4.30,9.27) |
|  | No | 417 | 112 | 1 | | 1 | |
| Smoking habit of male partner | Yes | 29 | 53 | 0.000 | 3.90(2.41, 6.29) | 0.089 | 1.64(0.93,2.91) |
|  | No | 486 | 228 | 1 | | 1 | |
| Family size | ≤ 3 | 199 | 66 | 1 | | 1 | |
|  | 4 to 5 | 185 | 115 | 0.001 | 1.87(1.30, 2.69) | 0.127 | 0.62(0.33,1.15) |
|  | ≥ 6 | 131 | 100 | 0.000 | 2.30(1.57, 3.37) | 0.778 | 1.07(0.68,1.69) |
| Presence of children | Yes | 418 | 258 | 0.000 | 2.60(1.61, 4.21) | 0.137 | 1.65(0.85,3.20) |
|  | No | 97 | 23 | 1 | | 1 | |
| Number of children | No | 97 | 23 | 1 | | - | |
|  | Single | 142 | 57 | 0.060 | 1.69(0.98, 2.93) | - | - |
|  | 2-4 | 269 | 193 | 0.000 | 3.03(1.85, 4.94) | - | - |
|  | ≥ 5 | 7 | 8 | 0.006 | 4.82(1.59,14.65) | - | - |
| Extended family living together | Yes | 233 | 154 | 0.010 | 1.47(1.10, 1.97) | 0.180 | 1.33(0.88,2.03) |
|  | No | 282 | 127 | 1 | | 1 | |

**Bivariate and multivariate logistic regression analyses of factors for Physical violence**

| **Variable** | **Category** | **Violence** | | **P-value** | **COR(95% CI)** | **P-value** | **AOR(95% CI)** |
| --- | --- | --- | --- | --- | --- | --- | --- |
|  |  | **No** | **Yes** |  |  |  |  |
| Age of women in years | 18 to 28 | 288 | 44 | 0.069 | 0.58(0.32, 1.04) | 0.332 | 1.70 (0.58, 4.97) |
|  | 29 to 38 | 310 | 58 | 0.238 | 0.71(0.40, 1.25) | 0.479 | 1.38(0.56, 3.41) |
|  | 39 to 45 | 76 | 20 | 1 | | 1 | |
| Woman Education | No formal educ. | 92 | 23 | 0.000 | 8.80(3.24,23.91) | 0.007 | 4.49(1.52,13.31) |
|  | Primary educ. | 169 | 47 | 0.000 | 9.79(3.80,25.21) | 0.001 | 5.66(2.05,15.65) |
|  | Junior educ. | 117 | 28 | 0.000 | 8.42(3.16,22.44) | 0.001 | 6.17(2.15,17.70) |
|  | Secondary educ. | 120 | 19 | 0.001 | 5.57(2.03,15.33) | 0.002 | 5.44(1.85,16.00) |
|  | Diploma & above | 176 | 5 | 1 | | 1 | |
| Own income of woman | No | 323 | 69 | 0.080 | 1.42(0.96,2.09) | 0.020 | 1.76(1.09, 2.84) |
|  | Yes | 351 | 53 | 1 |  | 1 |  |
| Age of male partner | 20 to 30 years | 216 | 30 | 0.070 | 0.60(0.34,1.04) | 0.512 | 0.71(0.26, 1.96) |
|  | 31 to 40 years | 338 | 64 | 0.404 | 0.81(0.50, 1.33) | 0.942 | 0.97(0.44, 2.15) |
|  | 41 to 68 years | 120 | 28 | 1 | | 1 | |
| Male partner Education | No formal educ. | 57 | 20 | 0.001 | 3.13(1.62, 6.03) | 0.096 | 2.00(0.89, 4.50) |
|  | Primary educ. | 160 | 39 | 0.005 | 2.17(1.27,3.74) | 0.750 | 1.12(0.57, 2.18) |
|  | Junior educ. | 106 | 17 | 0.286 | 1.43(0.74, 2.76) | 0.634 | 0.83(0.38, 1.81) |
|  | Secondary educ. | 128 | 21 | 0.228 | 1.46(0.79,2.72) | 0.791 | 0.91(0.44, 1.88) |
|  | Diploma & above | 223 | 25 | 1 | | 1 | |
| Religious belief of male partner | Weak | 24 | 6 | 0.213 | 1.85(0.70, 4.84) | 0.751 | 0.83(0.27, 2.58) |
|  | Medium | 399 | 82 | 0.057 | 1.52(0.99, 2.33) | 0.825 | 1.06(0.64, 1.76) |
|  | Strong | 251 | 34 | 1 | | 1 | |
| Alcoholics habit of male partner | Yes | 177 | 90 | 0.000 | 7.90(5.09,12.24) | 0.000 | 5.58(3.46, 8.99) |
|  | No | 497 | 32 | 1 |  | 1 |  |
| Smoking habit of male partner | Yes | 42 | 40 | 0.000 | 7.34(4.50,11.99) | 0.000 | 4.46(2.51, 7.95) |
|  | No | 632 | 82 | 1 | | 1 | |
| Family size | ≤ 3 | 236 | 29 | 1 | | 1 | |
|  | 4 to 5 | 259 | 41 | 0.328 | 1.29(0.78, 2.14) | 0.400 | 1.37(0.66, 2.82) |
|  | ≥ 6 | 179 | 52 | 0.001 | 2.36(1.44, 3.88) | 0.027 | 2.47(1.11, 5.53) |
| Presence of children | Yes | 566 | 110 | 0.082 | 1.75(0.93, 3.29) | 0.929 | 1.04(0.44, 2.44) |
|  | No | 108 | 12 | 1 | | 1 | |
| Number of children | No | 108 | 12 | 1 | | 1 | |
|  | Single | 178 | 21 | 0.875 | 1.06(0.50, 2.24) | - | - |
|  | 2 to 4 | 378 | 84 | 0.034 | 2.00(1.05, 3.80) | - | - |
|  | ≥ 5 | 10 | 5 | 0.016 | 4.50(1.32,15.37) | - | - |
| Extended family living together | Yes | 321 | 66 | 0.189 | 1.30(0.88, 1.91) | 0.669 | 1.12(0.66, 1.92) |
|  | No | 353 | 56 | 1 | | 1 | |

Bivariate and multivariate analysis of factors associated with sexual violence

| **Variable** | **Category** | **Violence** | | **P-value** | **COR(95% CI)** | **P-value** | **COR(95% CI)** |
| --- | --- | --- | --- | --- | --- | --- | --- |
|  |  | **No** | **Yes** |  |  |  |  |
| Residence | Rural | 103 | 12 | 0.127 | 0.61(0.33, 1.15) | 0.376 | 0.71(0.33, 1.53) |
|  | Urban | 572 | 109 | 1 | | 1 | |
| Age of women in years | 18 to 28 | 287 | 45 | 0.050 | 0.56(0.31,1.00) | 0.264 | 1.77(0.65,4.85) |
|  | 29 to 38 | 313 | 55 | 0.104 | 0.63(0.36,1.10) | 0.907 | 1.05(0.45,2.45) |
|  | 39 to 45 | 75 | 21 | 1 | | 1 | |
| Woman Education | No formal educ. | 87 | 28 | 0.000 | 4.53(2.20, 9.35) | 0.194 | 1.77(0.76, 4.17) |
|  | Primary educ. | 180 | 36 | 0.003 | 2.82(1.42, 5.59) | 0.377 | 1.43(0.65, 3.13) |
|  | Junior educ. | 115 | 30 | 0.000 | 3.67(1.81, 7.47) | 0.005 | 3.19(1.42, 7.14) |
|  | Secondary educ. | 124 | 15 | 0.188 | 1.70(0.77, 3.77) | 0.447 | 1.41(0.58, 3.44) |
|  | Diploma & above | 169 | 12 | 1 | | 1 | |
| Own income of woman | No | 355 | 49 | 0.015 | 1.63(1.10, 2.42) | 0.001 | 2.21(1.37, 3.57) |
|  | Yes | 320 | 72 | 1 | | 1 | |
| Relation status | Married | 504 | 80 | 0.051 | 1.51(0.99, 2.29) | 0.069 | 1.60(0.97, 2.65) |
|  | Unmarried | 171 | 41 | 1 | | 1 | |
| Women access to media | No | 47 | 18 | 0.004 | 2.34(1.31, 4.18) | 0.007 | 2.83(1.33, 6.02) |
|  | Yes | 628 | 103 | 1 | | 1 | |
| Pregnancy status | Pregnant | 85 | 20 | 0.240 | 1.37(0.81, 2.34) | 0.328 | 1.38(0.73, 2.61) |
|  | Not pregnant | 590 | 101 | 1 | | 1 | |
| Attitude of women on IPV | Acceptable | 13 | 12 | 0.000 | 5.61(2.49,12.61) | 0.000 | 7.35(2.76,19.62) |
|  | Not acceptable | 662 | 109 | 1 | | 1 | |
| Age of male partner | 20 to 30 years | 220 | 26 | 0.005 | 0.45(0.25, 0.79) | 0.050 | 0.39(0.15, 1.00) |
|  | 31 to 40 years | 238 | 64 | 0.168 | 0.72(0.44, 1.15) | 0.455 | 0.76(0.36, 1.58) |
|  | 41 to 68 years | 117 | 31 | 1 | | 1 |  |
| Male partner Education | No formal educ. | 50 | 27 | 0.000 | 7.34(3.72,14.47) | 0.000 | 4.90(2.13,11.25) |
|  | Primary educ. | 162 | 37 | 0.000 | 3.10(1.69, 5.70) | 0.387 | 1.40(0.65, 3.02) |
|  | Junior educ. | 105 | 18 | 0.018 | 2.33(1.16, 4.70) | 0.759 | 1.14(0.50, 2.60) |
|  | Secondary educ. | 127 | 22 | 0.012 | 2.35(1.21, 4.60) | 0.419 | 1.39(0.63,3.08) |
|  | Diploma & above | 231 | 17 | 1 | | 1 | |
| Male partner occupation | Farmer | 62 | 9 | 0.820 | 1.10(0.50, 2.39) | 0.161 | 0.49(0.18, 1.33) |
|  | Labor worker | 152 | 30 | 0.134 | 1.49(0.88, 2.51) | 0.554 | 0.82(0.41, 1.61) |
|  | Trader/Petit-trade | 182 | 45 | 0.010 | 1.86(1.16, 2.99) | 0.335 | 1.34(0.74, 2.40) |
|  | Govt/NGO-emp. | 279 | 37 | 1 | | 1 | |
| Religious belief of male partner | Weak | 19 | 11 | 0.000 | 6.92(2.93,16.37) | 0.018 | 3.41(1.24, 9.37) |
|  | Medium | 393 | 88 | 0.000 | 2.68(1.64, 4.38) | 0.001 | 2.46(1.42, 4.29) |
|  | Strong | 263 | 22 | 1 | | 1 | |
| Alcoholics habit of male partner | Yes | 205 | 62 | 0.000 | 2.41(1.63, 3.57) | 0.067 | 1.54(0.97, 2.46) |
|  | No | 470 | 59 | 1 | | 1 | |
| Smoking habit of male partner | Yes | 53 | 29 | 0.000 | 3.70(2.24, 6.12) | 0.003 | 2.48(1.35, 4.55) |
|  | No | 622 | 92 | 1 | | 1 | |
| Family size | ≤ 3 | 237 | 28 | 1 |  | 1 | |
|  | 4 to 5 | 254 | 46 | 0.095 | 1.53(0.93, 2.53) | 0.039 | 2.08(1.04, 4.17) |
|  | ≥ 6 | 184 | 47 | 0.003 | 2.16(1.30, 3.59) | 0.003 | 3.01(1.44, 6.29) |
| Presence of children | Yes | 567 | 109 | 0.088 | 1.73 (0.92, 3.25) | 0.461 | 1.40(0.57, 3.45) |
|  | No | 108 | 12 | 1 | | 1 | |
| Number of children | No | 108 | 12 | 1 | | - | |
|  | Single | 174 | 25 | 0.490 | 1.29(0.62, 2.68) | - | - |
|  | 2-4 | 384 | 78 | 0.066 | 1.83(0.96, 3.48) | - | - |
|  | ≥5 | 9 | 6 | 0.003 | 6.00(1.82,19.78) | - | - |
